# Popliteal Entrapment Syndrome as a Cause of Chronic Lower Extremity Pain in a 16-Year Old

**DOI:** 10.7759/cureus.13723

**Published:** 2021-03-05

**Authors:** Mackenzie N Naert, Brittany Glassberg, Daniel Han, Joseph Truglio

**Affiliations:** 1 Obstetrics and Gynecology, Brigham and Women's Hospital, Harvard Medical School, Boston, USA; 2 Internal Medicine-Pediatrics, Icahn School of Medicine at Mount Sinai, New York, USA; 3 Vascular Surgery, Icahn School of Medicine at Mount Sinai, New York, USA

**Keywords:** popliteal entrapment, med-peds, adolescent medicine

## Abstract

Popliteal entrapment syndrome is an uncommon cause of intermittent claudication in young patients lacking atherosclerotic risk factors. ZS is a 16-year-old cisgender female with type 1 diabetes complicated by microalbuminuria, obesity (body mass index (BMI) = 45.86 kg/m²), and a history of perinatal stroke with residual right-sided hemiparesis, who presented with six months of worsening bilateral, exertional lower extremity pain. Common causes of chronic bilateral lower extremity pain include peripheral vascular disease and diabetic neuropathy. Less common etiologies include trauma, infection, or juvenile idiopathic arthritis. Given her risk factors, the patient's pain was initially managed as a diabetic neuropathy with pregabalin. Symptoms failed to improve, and she re-presented with positional coolness of the right lower extremity, diminished pulses of the bilateral lower extremities, and weakness in her toes. CT angiography demonstrated occlusion of the right distal superficial femoral and popliteal arteries and diffused tibial disease. Ultimately, the patient was discovered to have right-sided femoral-popliteal occlusion, and she required urgent femoral-tibial bypass. Despite an initial improvement in symptoms postoperatively, she continued to have lower extremity pain and recurrent arterial thrombi, even with antiplatelet and anticoagulation therapy. Eventually, the patient required a right-sided below the knee amputation. This case highlights the high index of suspicion that clinicians must have in young patients with lower extremity pain, both with and without atherosclerotic risk factors, as early intervention facilitates better outcomes. Introduction

## Introduction

Popliteal entrapment syndrome is an uncommon cause of intermittent claudication in younger patients lacking atherosclerotic risk factors. The estimated incidence of the diagnosis is 0.17-3.5% [1]. For this reason, popliteal entrapment syndrome is often a delayed diagnosis [2]. Here we present the case of an adolescent diagnosed with popliteal entrapment syndrome after being initially diagnosed and treated for diabetic neuropathy in the setting of poorly-controlled type 1 diabetes. This case demonstrates the importance of having a broad differential for claudication in a young adult.

## Case presentation

Patient Information

**Figure 1:**
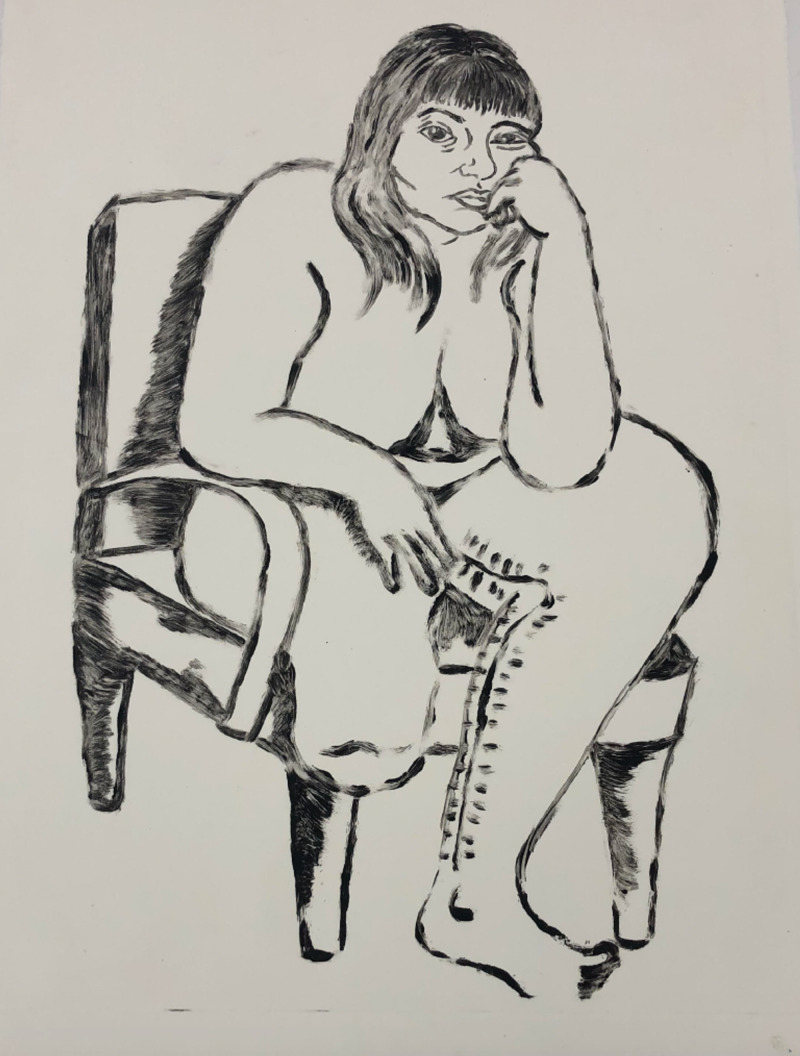
Self-portrait of ZS created during post-operative period

ZS is a 16-year-old female with Type 1 diabetes diagnosed at age 11, complicated by microalbuminuria, obesity (body mass index (BMI)= 45.86kg/m²), and perinatal history of stroke with residual right-sided hemiparesis, who presented with six months of worsening bilateral, exertional lower extremity pain. She described a burning pain associated with numbness and tingling, worse with ambulation and when supine. The pain was progressive and resulted in difficulty walking and multiple school absences. The patient has a history of depression with cutting behaviour; she denies suicidal and homicidal ideations. Family history is significant for type 1 diabetes mellitus and Hashimoto's thyroiditis in her younger sister and her mother's coronary artery disease. Social history is significant for recent tobacco use initiation (1-4 cigarettes per day) and marijuana use (1-2 times per week). She denies alcohol consumption. The patient uses art as a mechanism for coping with significant pain and medical conditions (Figure 1). The patient's medication regimen consists of a continuous insulin pump, Metformin 500 mg daily, and Duloxetine 60 mg daily.

Clinical Findings

On initial presentation, the patient's vital signs were within normal limits. Her BMI was 45.86kg/m². She was a well-appearing, obese adolescent in no acute distress. Physical exam was notable for intact pedal pulses bilaterally and intact sensation to light touch and vibration in both feet. Her right first toe was erythematous and edematous with onychocryptosis. There were early bilateral stage 1 linear heel ulcers. Her neurological exam was significant for the distal flaccid weakness of the right hand and foot with an upgoing Babinksi on the right, consistent with perinatal stroke history. There was no muscular atrophy or fasciculation. She had a right circumducting hemiparetic gait.

The patient's initial labs were significant for an elevated Hemoglobin A1c (HbA1c) 8.9% (normal <6%), thyroid-stimulating hormone (TSH) level within normal limits (2.96mIU/L; reference 0.55-4.78), and Vitamin B12 level within normal limits (327ng/mL; reference 211-911ng/mL). See Table 1 for a comprehensive list of laboratory evaluations. She was referred to neurology for neuromuscular and electrophysiological evaluation and was found to have a distal sensory axonal polyneuropathy. Given the elevated Hemoglobin A1c and her sensory polyneuropathy, she was initially thought to have diabetic neuropathy and managed as such.

**Table 1:** Laboratory evaluation in search for etiology of popliteal entrapment syndrome H: high, INR: international normalized ratio, C-Anca: C-Antineutrophil cytoplasmic antibodies, P-Anca: P-Antineutrophil cytoplasmic antibodies, Ab: antibodies, Anti-TPO Ab: anti-thyroid peroxidase antibodies, Anti-human TTG-IgA: Anti-human tissue transglutaminase immunoglobulin A, IgG: Immunoglobulin G, IgM: Immunoglobulin M

Hematologic & Endocrinologic Studies			Rheumatologic Studies		
Component	Value	Normal	Component	Value	Normal
D-dimer	1.04 *μg/mL *(H)	<0.50 *μg*/*ml*	C Reactive Protein HS	18.2 mG/L (H)	0.0-5.0 mG/L
Prothrombin Time	13.6s	11.8-14.3 s	Erythrocyte Sedimentation Rate	29 mm/hour (H)	0-15 mm/hour
INR	1.1	0.9-1.1	Cystatin C	0.93 mg/L	0.50-1.00 mg/L
Activated Partial Thromboplastin Time	39.1 s (H)	25.0-35.0s	Anti-human TTG-IgA	1 mg/dL	<4 mg/dL
T4 (Thyroxine) Total	7.1 mcg/dL	5.7-11.4 mcg/dL	ANA	Negative	Negative
T4 (Thyroxine) Free	1.06 mcg/dL	0.80-1.76 mcg/dL	Lupus Anticoagulant	Negative	Negative
Thyroglobulin Ab	<20.0 IU/ml	0-40.0 IU/ml	Anti-Thrombin III	66% (L)	81-113%
Anti-TPO Ab	20.6 IU/ml	0-35.0 IU/ml	Factor V Leiden	Negative	Negative
Thyroid Stimulating Hormone	2.959 uIU/mL	0.34-5.60 uIU/mL	C-Anca	Negative	Negative
Vitamin B12	327 ng/mL	211-911 ng/mL	P-Anca	Negative	Negative
Homocysteine	6.3 umol/L	5.0-15.0 umol/L	Antiphospholipid IgG	7 units	0-14 units
Protein C Activity	121 IU/dL	75-133 IU/dL	Antiphospholipid IgM	24 (H) units	0-14 units
Protein S Activity	52 IU/dL	52-151 IU/dL	MTHFR C677T/A1298C Mutation	Negative	Negative

**Figure 2:**
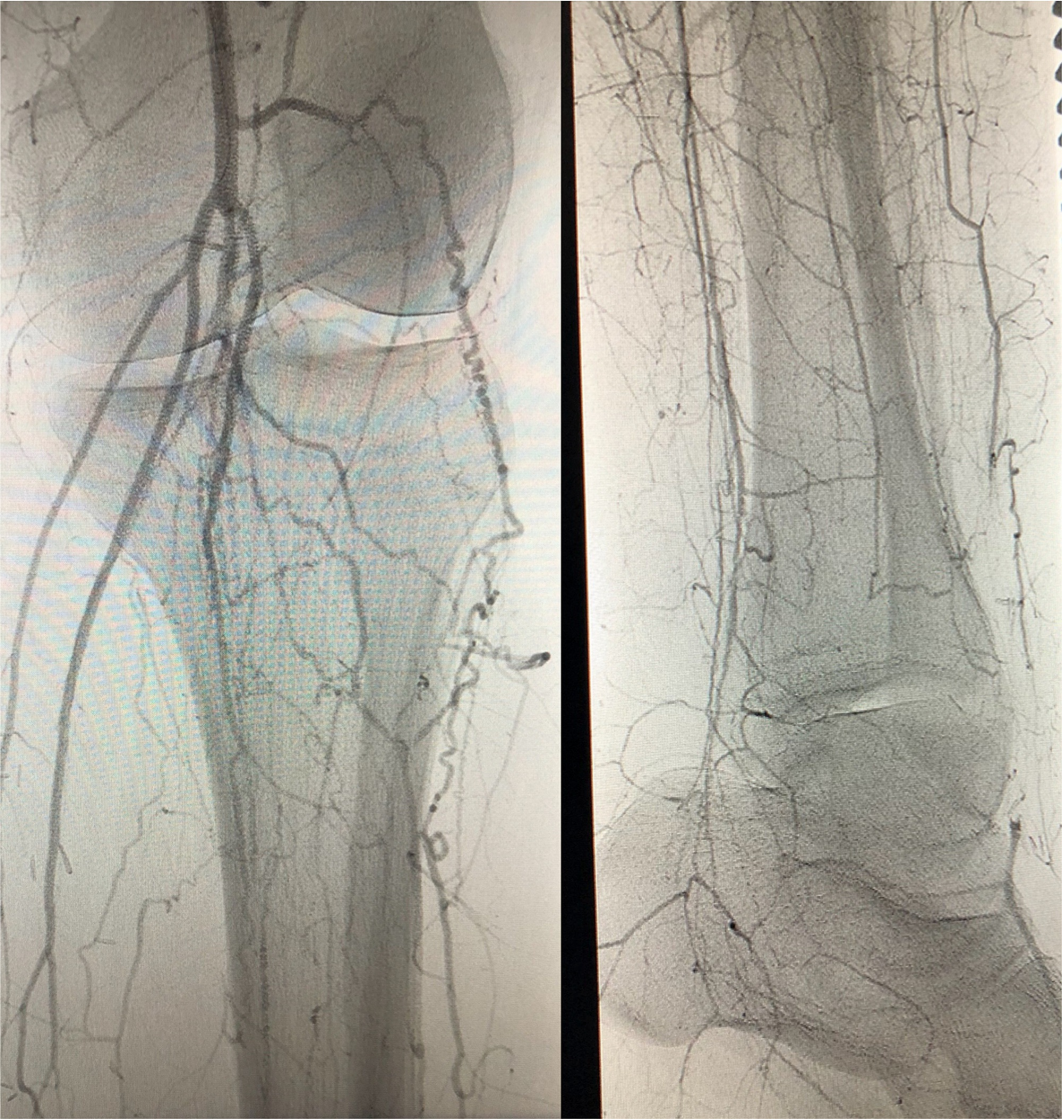
Right Lower Extremity CT Angiogram April 2017 Left: Right distal superficial femoral and popliteal artery collateral circulation development. Right: Right lower extremity collateral circulation to a level of dorsalis pedis artery.

She was prescribed Pregabalin 100mg BID alleviate pain, to which she had minimal response. One month later, she presented with positional coolness in her right lower extremity (see Table 2 for specific timeline). On exam, she was found to have diminished pulses in bilateral lower extremities and difficulty moving her toes. Her extremities were warm, the sensation was intact, and the neurological exam was grossly intact. Her brachial ankle index at the time of admission was 0.35 right lower extremity and 0.45 left lower extremity. She was found on computerized tomography (CT) angiography to have occlusion of the right distal superficial femoral and popliteal arteries and diffuse tibial disease (Figure 2).

**Table 2:** Timeline of events and interventions CT: Computerized tomography, HbA1c: Hemoglobin A1c, TSH: Thyroid-stimulating hormone

Relevant Past Medical History and Interventions			
ZS is a 16-year-old female with Type 1 diabetes diagnosed at age 11 complicated by microalbuminuria, obesity (BMI = 45.86 kg/m²), and a history of prenatal stroke with residual right-sided hemiparesis, who presented with 6 months of worsening bilateral, exertional lower extremity pain.			
Date	Presentation & Evaluation	Diagnostic Testing & Results	Interventions
August 20th, 2015	Initial presentation to the Emergency Department at an outside hospital for pain with ambulation x 6 months	HbA1c (elevated at 8.9%) B12 (within normal limits at 327 ng/mL) TSH (within normal limits at 2.96 mIU/L)	Neurology Referral
October 15th, 2015	Referral to neurology for presumed diabetic neuropathy	Electrophysiologic testing consistent with distal sensory axonal polyneuropathy	Pregabalin 100mg BID
October 28, 2015	Presented to the Emergency Department with first episode of acute ischemia. Admitted to Pediatric Intensive Care Unit.	CT angiography demonstrating right-sided arterial thrombus	Semi-urgent Popliteal Bypass Surgery: Right superior femoral artery to proximal anterior tibial artery bypass and embolectomy of the bypass
November 2015- January 2017	Presented to Emergency Department for recurrent claudication	CT angiogram demonstrates recurrent bilateral thrombi	Multiple revascularization procedures including bypass surgeries (6), embolectomies/ thrombectomy (2), thrombolysis, percutaneous transluminal angioplasty & stent placement
May 5th, 2017	Presented to Emergency Department for continued recurrent claudication	CT angiogram demonstrates occluded/failed femoral artery-anterior tibial bypass	Right below the knee amputation. Follow-up search for etiology

The patient was taken to the operating room for a thromboembolectomy and right superior femoral artery to proximal anterior tibial artery bypass. Over the next 18 months, the post-operative course was complicated by recurrent bilateral thrombi requiring six popliteal bypass surgeries and two embolectomies. Subsequent workup was unrevealing for a hypercoagulable state (including negative ANA, lupus anticoagulant, antithrombin III activity, and Factor V Leiden mutations) or vasculitis (including negative c-ANCA and p-ANCA; see Table 2). Despite an initial improvement in symptoms, she continued to have recurrent thrombosis of her bypass even with antiplatelet and anticoagulation therapy, worsening ischemic rest pain and foot ulcerations, ultimately requiring a right-sided below the knee amputation. Medical management postoperatively was focused on clot-prevention with aspirin 81mg and rivaroxaban 20mg daily. Her pain was managed with gabapentin 600mg BID and methadone 2.5mg BID. Her recovery was complicated by chronic pain syndrome and delayed wound closure requiring multiple debridements. The patient is currently awaiting prosthesis placement, and she is engaged in trauma-informed care, a comprehensive therapeutic approach centred on supporting patients as they process adverse experiences [3].

## Discussion

Popliteal entrapment syndrome is a rare condition in which the tendons and muscles near the knee compress the popliteal artery, restricting blood flow to the lower leg [2,4]. The incidence of popliteal entrapment syndrome is likely underestimated due to underdiagnosis, but recent reports estimate between 0.17% and 3.5% [2]. The condition is most commonly caused by a congenital anomaly that causes the medial or lateral gastrocnemius head to shift towards the popliteal artery during knee flexion, compressing the artery [2,4-6]. It is most commonly seen in young men (85% of cases are male) [2], particularly in bicyclists, runners, and active military personnel (60% of cases occur in male athletes < 30 years old) [2]. It is hypothesized that repetitive overuse gradually leads to the thickening of vessel walls in the popliteal artery due to excessive pressure from the muscle belly [6].

The embryonic development of the structures of the popliteal fossa accounts for the anatomic variability of this diagnosis. Both the popliteal artery and medial head of the gastrocnemius muscle develop at the same embryological time point. Anomalies relating to the popliteal artery's position, medial head of the gastrocnemius and popliteus muscles can account for the etiology of this syndrome [2]. Despite these anatomic variants, many people born with this disease's congenital form are often asymptomatic, suggesting a multifactorial etiology of the disease. It is thought that changes related to the gastrocnemius muscle, particularly while running or marching, cause muscle hypertrophy with external compression and impingement of the popliteal artery [2]. Notably, unlike most arterial occlusion causes, popliteal entrapment syndrome is not associated with an increased risk of cardiovascular disease [5-7].

While most patients do not have clinically significant disease, those who do have symptoms typically report deep calf pain following intense exercise involving ankle dorsiflexion and plantarflexion, resembling claudication, usually within the 2nd decade of life. Further, patients may experience paresthesias across the proximal posterior calf during exercise, followed by pale, discoloured feet and toes. Physical examination is usually normal, with possible hypertrophy of the calf muscles [2,4,5]. Chronic repeated arterial compression can lead to acute thrombus formation and a presentation with acute limb-threatening ischemia, as was the case for ZS [2]. This case is unique because the patient presented here is both female and a non-athlete, different from the majority of patients with the disease. Perhaps an unknown or multifactorial etiology, including her history of right-sided paresis and family history of CAD, exists in our patient and others like her.

Workup for popliteal entrapment syndrome includes assessing distal pulses and ankle-brachial index, followed by Doppler ultrasound and compartmental pressure measurement, with an option for CT angiogram and MRI. Confirmed diagnosis of popliteal entrapment requires demonstration of abnormal muscle attachments on imaging; without this result, it is referred to as functional popliteal entrapment syndrome [1,2]. Newer studies highlight the advantage of intravascular ultrasound in diagnosis, as this novel imaging modality allows for specific localization of compression and can assist in operative planning.

Current recommendations for management include surgical myotomy or botulinum toxin A injections. By the time many patients seek medical care, they are typically highly symptomatic, and the preferred management involves decompression of the entrapped artery by the release of abnormal muscle connections. Arterial reconstruction or revascularization is only performed if there is evidence of arterial injury or thrombosis. The preferred surgical therapy is decompression of the entrapped artery and reconstruction with vein graft interposition [2,4,5]. Thromboendarterectomy is the second line [7,8].

## Conclusions

This case highlights the high index of suspicion that clinicians must have in young patients with claudication. The majority of patients with popliteal entrapment syndrome present late in the disease course, and ZS was diagnosed after 16 months of symptoms. The strongest predictive factor for successful treatment of popliteal entrapment syndrome is early diagnosis and timely management, underscoring the importance of recognizing symptom subtleties in this disease.
